# Supplementary material for: Transcriptome Analysis of Induced Pluripotent Stem Cells and Neuronal Progenitor Cells, Derived from Discordant Monozygotic Twins with Parkinson’s Disease
Source: Cells. 2021 Dec 9;10(12):3478. doi: 10.3390/cells10123478 (PMC8700621; doi:10.3390/cells10123478)
Supplement: Supplementary file 1 [file cells-10-03478-s001.zip › Supplementary_text.pdf]

Supplementary text S1

Cell culture characterization

Normal karyotype of IPSC was confirmed (fig. 1) and fluorescent antibody staining was performed to confirm IPSC potential to differentiate into three germ layer derivates (fig. 1a).

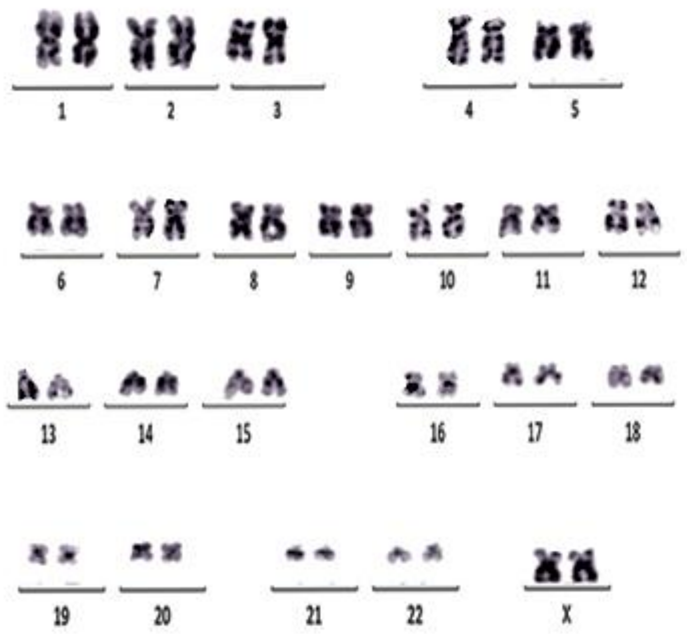

Figure 1a. Representative photo of normal karyotype of IPSC, derived from twin with PD

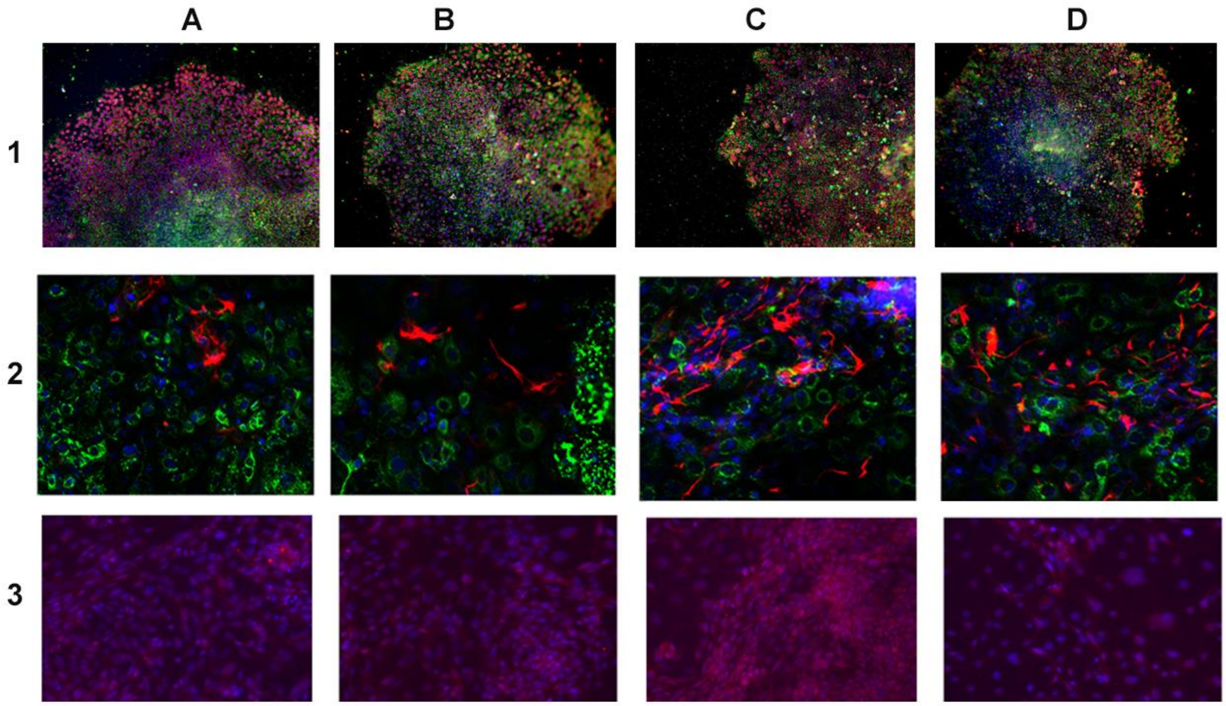

Figure. 1b
Immunocytochemical staining of IPSC for pluripotency markers
A, B – IPSC derived from healthy twins, C, D – IPSC derived from twins with PD.
1 - antibodies to Oct4 (red), SSEA4 (green), DAPI (blue)
2 - antibodies to entodermal marker AFP (red), mesodermal marker desmin (green)
3 — ectodermal marker Sox1 (purple), DAPI (blue). x100

Obtained NPC cultures were stained for neuron-specific Sox1 marker (fig. 1b). Fraction of all Sox1-positive cells was no less than 79% in all cases.

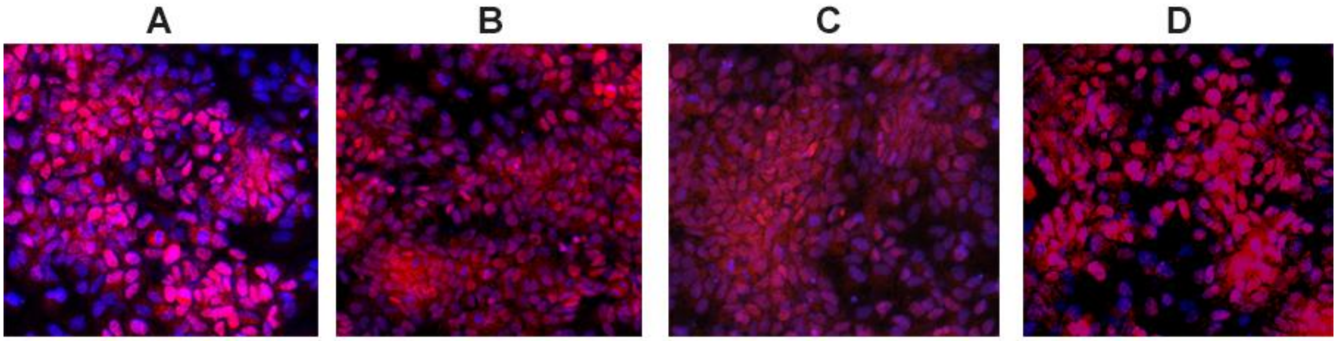

Fig. 1c
Immunocytochemical staining of NPC for neuronal marker Sox1
A, C – NPC derived from healthy twins, B.D. - NPC derived from twins with PD. x100

Supplementary text S2.

Expression of Table 4 genes by sample.

In order to investigate if the differential expression of genes in table 4 is driven by a single differentiation or cell culutres derived from single twins, we have created barplots (fig. 2a-f) with FPKM for those genes by sample. In no cases the difference appears to be driven by a single differentiation or a single twin.

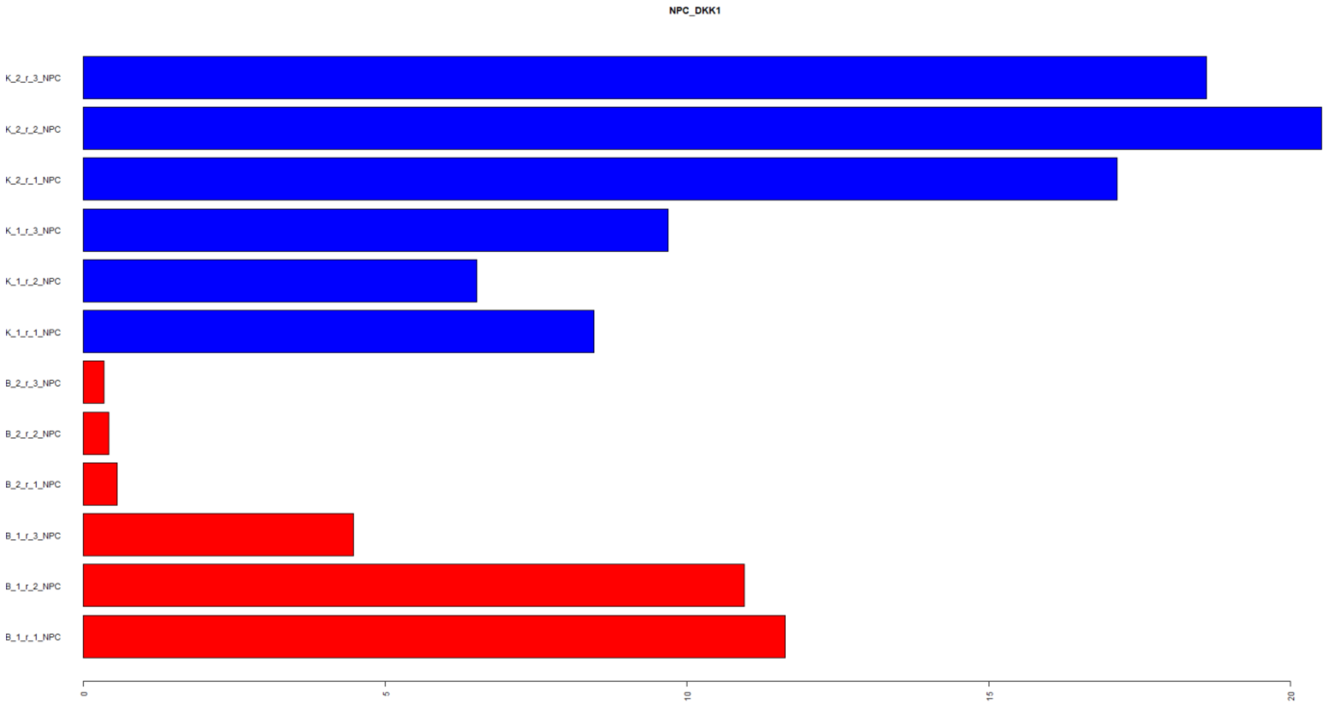

Figure 2a. Normalized counts (FPKM) of DKK1 expression in NPC cell lines. Here and in all following figures "K" and "B" letters and blue and red color notate control twins and twins with PD respectively, first number notates the pair of twins and second number notates a separate differentiation of cells.

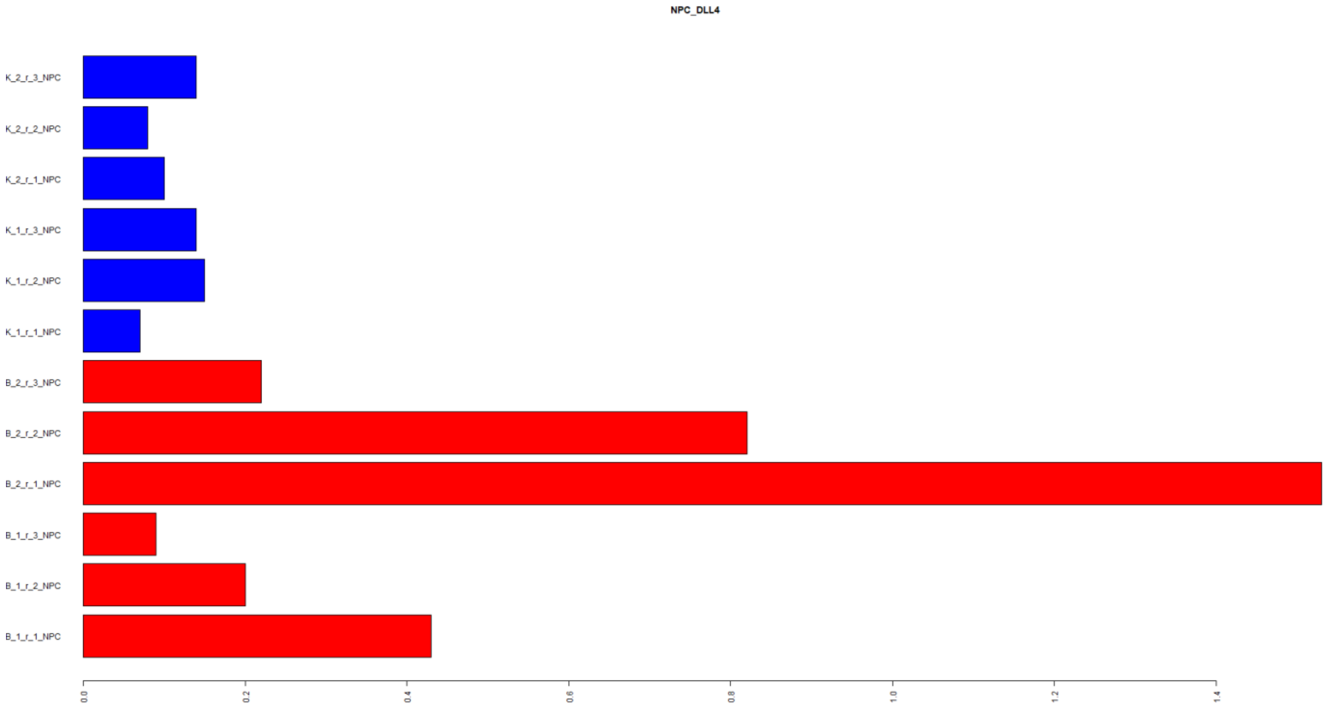

Figure 2b. Normalized counts (FPKM) of DLL4 expression in NPC cell lines.

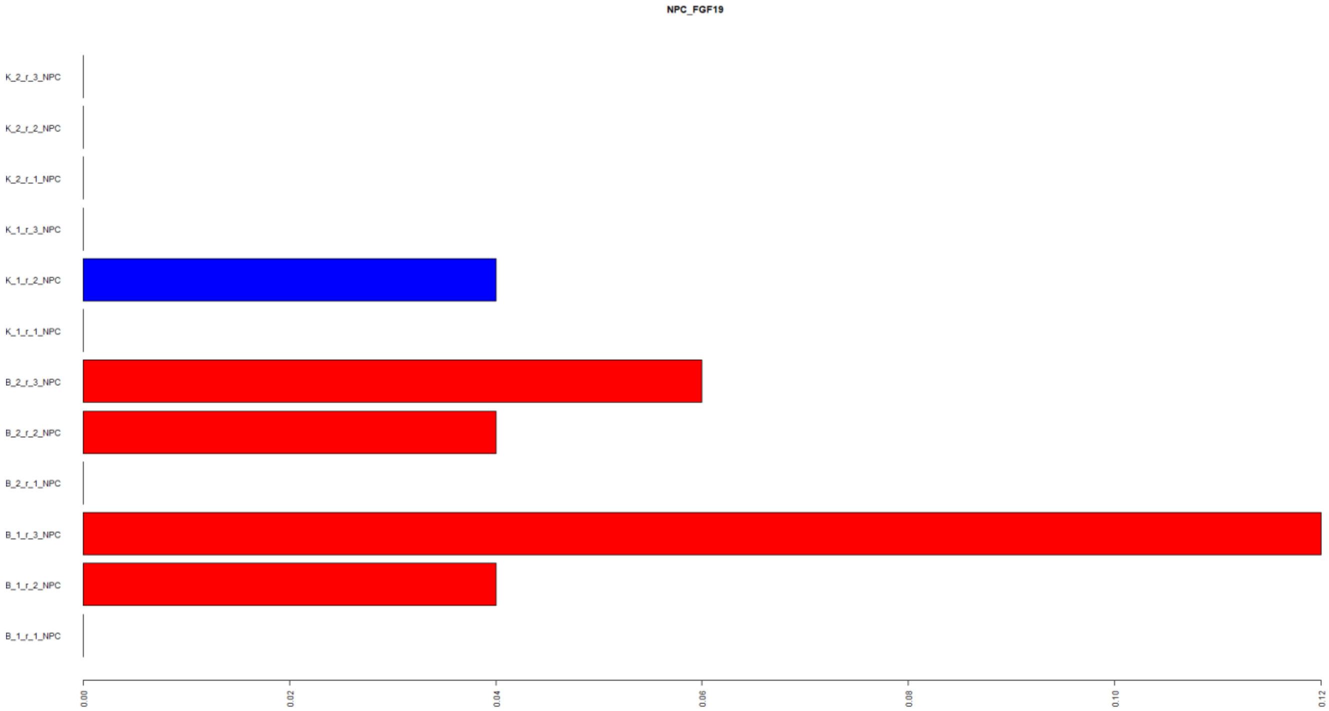

Figure 2c. Normalized counts (FPKM) of FGF19 expression in NPC cell lines.

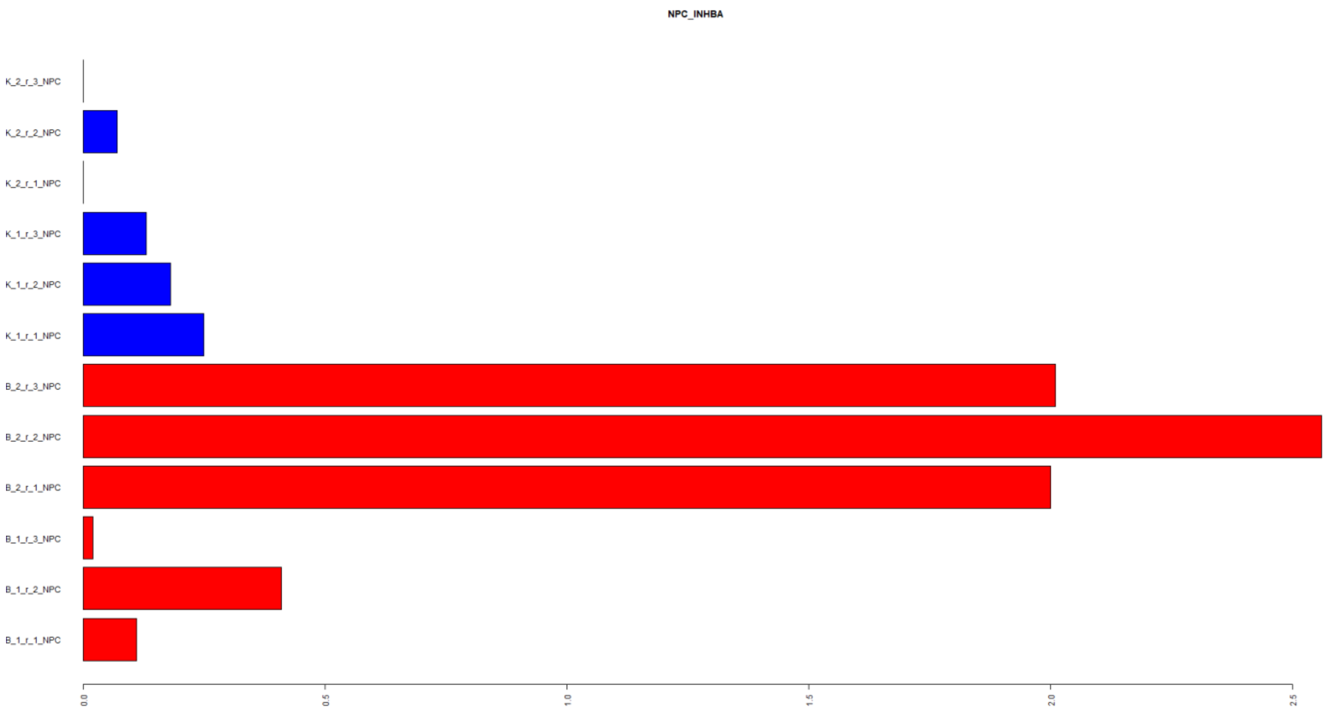

Figure 2d. Normalized counts (FPKM) of INHBA expression in NPC cell lines.

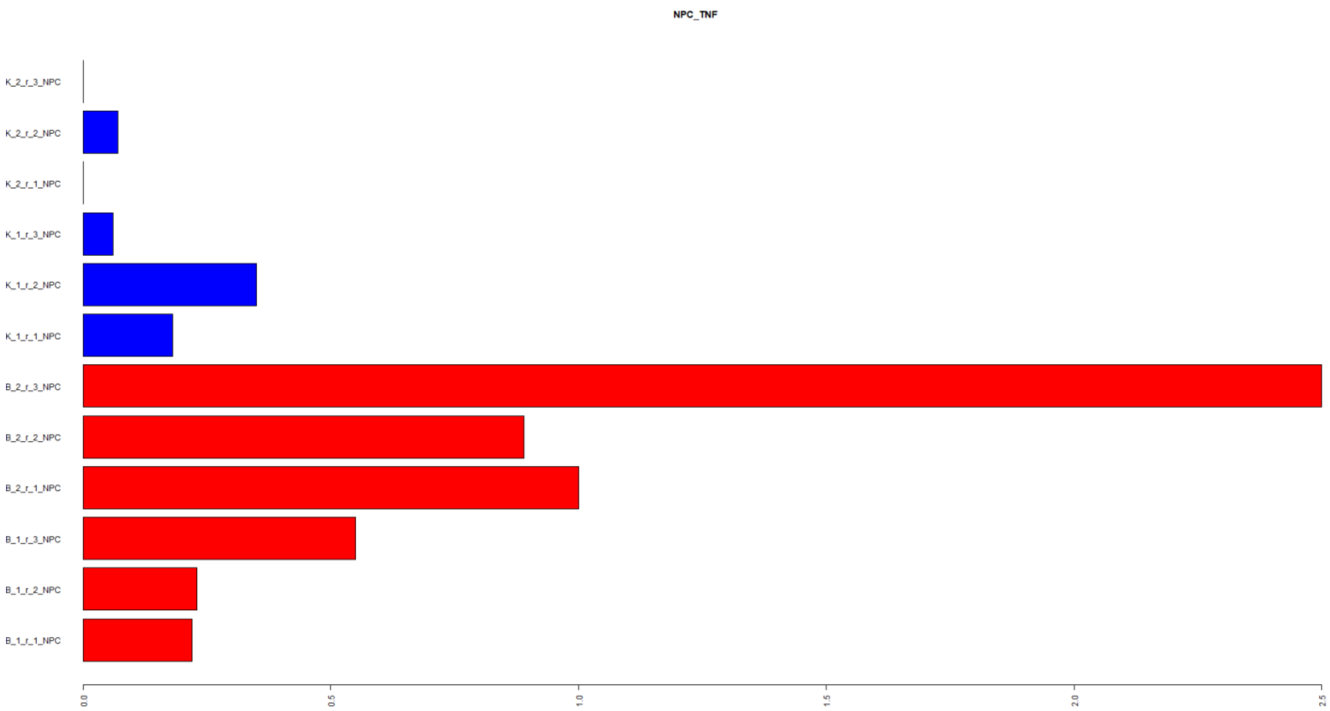

Figure 2e. Normalized counts (FPKM) of TNF expression in NPC cell lines.

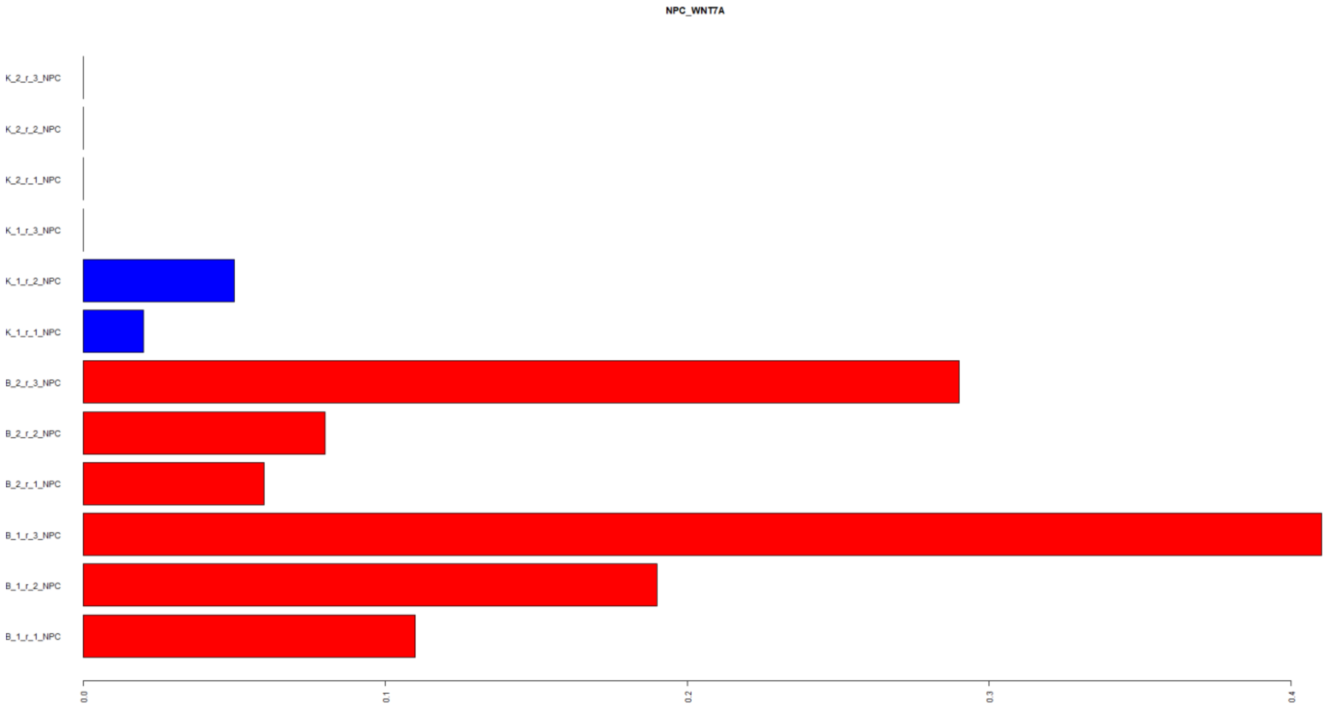

Figure 2f. Normalized counts (FPKM) of WNT7A expression in NPC cell lines
