# Supplementary figures and images for: Transcriptome Analysis of Induced Pluripotent Stem Cells and Neuronal Progenitor Cells, Derived from Discordant Monozygotic Twins with Parkinson’s Disease
Source: Cells. 2021 Dec 9;10(12):3478. doi: 10.3390/cells10123478 (PMC8700621; doi:10.3390/cells10123478)

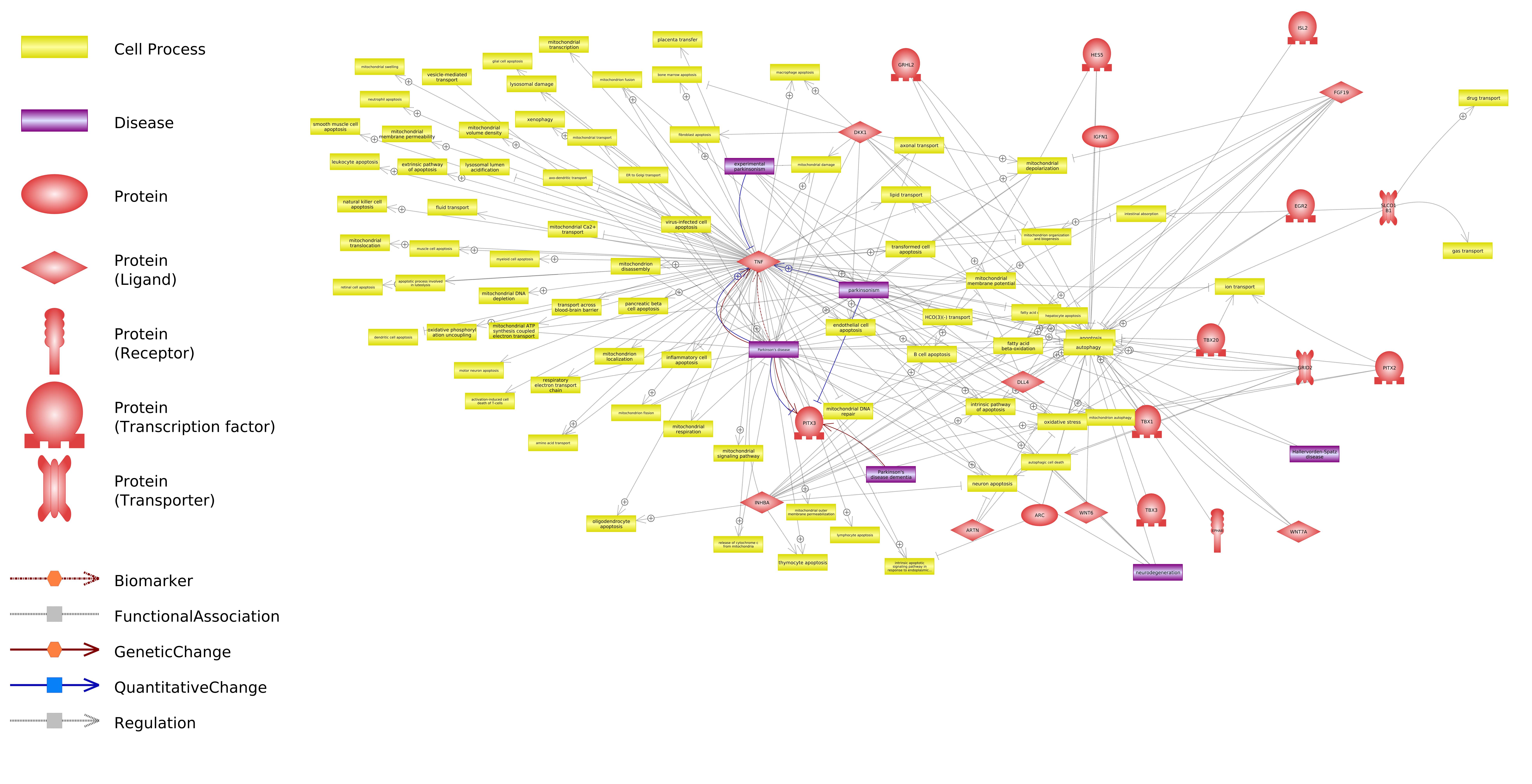

Supplement: Supplementary file 1 [file cells-10-03478-s001.zip › Supplementary_figure_S1.jpg]
